# Measuring Anxiety-Like Behaviors in Rodent Models of Traumatic Brain Injury

**DOI:** 10.3389/fnbeh.2021.682935

**Published:** 2021-10-29

**Authors:** Laura B. Tucker, Joseph T. McCabe

**Affiliations:** ^1^Preclinical Behavior and Models Core, F. Edward Hébert School of Medicine, Uniformed Services University of the Health Sciences, Bethesda, MD, United States; ^2^Department of Anatomy, Physiology and Genetics, F. Edward Hébert School of Medicine, Uniformed Services University of the Health Sciences, Bethesda, MD, United States

**Keywords:** anxiety, brain injury, behavior, common data elements, open field, light-dark box, elevated plus maze, elevated zero maze

## Abstract

Anxiety is a common complaint following acquired traumatic brain injury (TBI). However, the measurement of dysfunctional anxiety behavioral states following experimental TBI in rodents is complex. Some studies report increased anxiety after TBI, whereas others find a decreased anxiety-like state, often described as increased risk-taking behavior or impulsivity. These inconsistencies may reflect a lack of standardization of experimental injury models or of behavioral testing techniques. Here, we review the most commonly employed unconditioned tests of anxiety and discuss them in a context of experimental TBI. Special attention is given to the effects of repeated testing, and consideration of potential sensory and motor confounds in injured rodents. The use of multiple tests and alternative data analysis methods are discussed, as well as the potential for the application of common data elements (CDEs) as a means of providing a format for documentation of experimental details and procedures of each published research report. CDEs may improve the rigor, reproducibility, as well as endpoint for better relating findings with clinical TBI phenotypes and the final goal of translation. While this may not resolve all incongruities in findings across laboratories, it is seen as a way forward for standardized and universal data collection for improvement of data quality and sharing, and advance therapies for neuropsychiatric symptoms that often present for decades following TBI.

## Introduction

Anxiety disorders are characterized by the DSM-V (Diagnostic and Statistical Manual of Mental Disorders, version 5) as excessive fear and anxiety with related behavioral disturbances (American Psychiatric Association, 2013), and are associated with comorbid conditions such as cardiovascular disease, migraine, hypertension, and gastrointestinal disease. Psychiatric disturbances including anxiety often persist for many years following traumatic brain injury (TBI) (Koponen et al., 2002, 2006; Scholten et al., 2016), a worldwide growing healthcare burden (James et al., 2019). A recent review pooled long-term prevalence of anxiety following TBI to be 36% (Scholten et al., 2016), and the presence of anxiety 10 years post-TBI has been found to be a strong predictor of poor psychosocial function (Draper et al., 2007).

Animal models have long played a role in the study of behavioral symptoms and pathophysiology following traumatic brain injury, as well as in the development of therapeutic agents for those maladies, albeit with limited success. Behavioral testing following experimental TBI in rodents has provided extensive data in multiple domains of behavior, including motor, cognitive, and neuropsychiatric function (File, 2001; Fujimoto et al., 2004; Malkesman et al., 2013; Osier et al., 2015). Although less attention is given to neuropsychiatric function than to motor and cognitive issues in translational research, the study of anxiety-like behaviors in animal models of TBI is popular due to their high clinical relevance (Scholten et al., 2016). The purpose of this review is to introduce the reader to the most common tests for anxiety employed following experimental TBI, followed by a discussion of the shortcomings and inconclusive results obtained from anxiety testing in animal models of TBI to date. Further attention will be given to potential testing confounds, such as sensory deficits and differences in overall activity levels. We conclude with an emphasis on suggestions for alternative methods of data analysis and the promise of common data elements (CDEs) as a means of improving preclinical studies translation.

## Unconditioned Tests of Anxiety in Rodents

Anxiety has been described in animals as a generalized psychological, physiological, and behavioral state induced by exposure to an unknown threat or internal conflict (Steimer, 2002, 2011). There are numerous tests for measuring anxiety-like behaviors in rodent models, and these assays are most often classified as “conditioned” and “unconditioned” tests (Bourin et al., 2007). Conditioned tests depend on the development of a conditioned response to an aversive stimulus, such as fear potentiated startle and conditioned defensive burying. It is important to note that performance on conditioned tests is often dependent on intact cognitive and sensory function, and as such may not be appropriate in a TBI setting if significant deficits are present. Unconditioned tests rely on spontaneous, natural responses to ethologically relevant, stressful situations, which take advantage of the conflict inherent in approach-avoidance situations. The conflict results in a competition between spontaneous exploratory behavior and the innate aversion of open, illuminated areas (Lister, 1990). Although they are not without criticism, unconditioned tests are the most frequently used assays in anxiety and TBI research, largely due to their ease of use. In this section we review the most popular unconditioned tests of anxiety; the reader is also directed to several comprehensive reviews (Lister, 1990; Belzung and Griebel, 2001; Castanheira et al., 2018; Harro, 2018).

### Open Field Test

**FIGURE 1:**
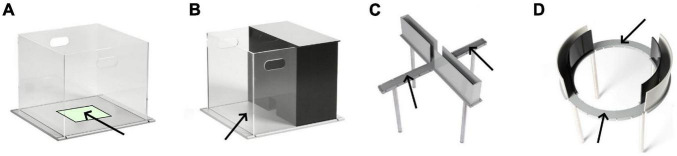
Common apparatuses for measuring unconditioned anxiety-like behaviors in rodents: **(A)** Open field, **(B)** Light-dark box, **(C)** Elevated plus maze, **(D)** Elevated zero maze. Arrows point to anxiogenic (lit and exposed) regions of the apparatus. Images from Stoelting, Co.

The open field test (OFT) (Figure 1A) was developed by Hall in 1934 and has become one of the most popular behavioral tests in multiple species. It is simple to perform and requires only basic equipment (Hall, 1934; Lister, 1990; Prut and Belzung, 2003). In the OFT, animals are individually placed in the center of a circular or rectangular/square arena [e.g., 40 cm × 40 cm for mice (Yu et al., 2012; Tucker et al., 2017); 100 cm × 100 cm for rats (McAteer et al., 2016; Tsuda et al., 2020)] with walls high enough to prevent escape, and behavior is recorded, typically with automated software. Trial lengths are as short as 2 min and up to an hour (Castanheira et al., 2018). When placed in the OF environment, rodents typically display thigmotactic behavior, staying near the walls of the apparatus and spending less time in the center, more exposed region. The most common measures analyzed include total horizontal distance traveled, number of rearing episodes [see (Sturman et al., 2018) for discussion], freezing, number of fecal boli, and proportion of time spent in a software-defined center zone of the apparatus (Crawley, 2007).

There has been a historical assumption that lower levels of ambulation in the OFT represent increased “emotionality” or anxiety (Hall, 1934; Lister, 1990). However, non-anxiolytic pharmacological agents can increase activity in the arena (Cunha and Masur, 1978), and general locomotor activity has been suggested to be “an unsuitable index of anxiety in psychopharmacological research” (Lister, 1990). Presently, the most commonly employed measure of anxiety is thigmotaxis (Seibenhener and Wooten, 2015), or alternatively, activity in the center region of the OF (either time spent in the center or distance traveled (McAteer et al., 2016; Tucker et al., 2017). Distance traveled in the center should be expressed as a percent of the total distance traveled if there are baseline differences in activity between experimental groups. Greater time spent or distance traveled in the center is interpreted as reduced or less anxiety. However, common anxiolytic drugs do not increase the amount of time spent in the center (Thompson et al., 2015), and the test has been criticized for its inability to dissociate locomotion, exploration, and anxiety (File, 2001). Thus, it has been suggested that conclusions regarding anxiety states in the OFT be considered only preliminary, and followed up with more specific anxiety tests such as the light-dark box or elevated plus maze (Crawley, 2007).

### Light-Dark Box

The light-dark box (LDB) (Figure 1A) is a modification of the OFT in which approximately two thirds of the apparatus is open, uncovered and brightly illuminated (e.g., ∼400 Lux), and the remaining one third is enclosed with darkened walls, and covered (Crawley and Goodwin, 1980; Bourin and Hascoet, 2003). An opening in the wall separating the two chambers allows the animal to move freely within the apparatus, and also allows a small amount of light to enter the darkened chamber resulting in a more natural setting. As rodents typically live in small tunnels, they will prefer the smaller darkened chamber, but are also driven by an urge to explore and thus also spend time in the larger brightened portion of the apparatus. The animal is placed in the illuminated section of the apparatus and monitored for 10 min; time spent in each chamber is recorded as well as the number of transitions between the dark and light compartments (as an index of activity). Increases in the time spent in the brightened compartment are suggested to reflect anxiolytic activity. Other parameters have been proposed including rearing, the latency to enter the dark compartment, amount of activity in each chamber (expressed as a function of time in each chamber), number of times the animal “peeks” from the dark chamber into the light but then retreats (Hascoët and Bourin, 1998; Bourin and Hascoet, 2003). However, after a careful review of the literature, Hascoët and Bourin concluded that the most reliable measure for assessing anxiolytic-like activity was the time spent in the brighter chamber (with greater time in the dark chamber representing anxiety-like behavior) (Hascoët and Bourin, 1998); this parameter provided a stable baseline and consistent dose-response increases in response to anxiolytic agents.

### Elevated Plus Maze and Elevated Zero Maze

The elevated plus maze (EPM; Figure 1C) is the most widely employed test for assessing anxiety-like behaviors and testing putative anxiolytic agents in rodents (Haller et al., 2013). The EPM was developed and pharmacologically validated in rats (Pellow et al., 1985), and quickly adapted for use in mice (Lister, 1987). The EPM is a plus-shaped apparatus, with two darkened and enclosed (but uncovered) arms and two exposed arms joined by a central square, elevated 50–100 cm above the floor. Compared to the OFT and LDB, the anxiogenic stimulus of the EPM is the absence of walls or thigmotactic cues in the open arms (rather than height) (Treit et al., 1993). Animals are individually placed in the center square, and activity is tracked for 5 min. Measures of anxiety include time spent in the open arms (expressed as a percent of total arm time) and number of entries into open arms (expressed as a percent of total arm entries) (Rodgers et al., 1997). Rodents with greater levels of anxiety will spend less time in (and make fewer entries into) the open exposed arms; these measures are sensitive to some anxiolytic agents (Pellow et al., 1985; Lister, 1987). An appropriate index of locomotor activity has been the subject of debate (Weiss et al., 1998).

The elevated zero maze (EZM; Figure 1D) is a modification of the EPM that is annular and has alternating “open” and “closed” quadrants (Shepherd et al., 1994). The EZM has the benefit of removing the center square, allowing simpler analysis of activity in open and closed regions of the apparatus. Interpretation of activity in the center square of the EPM, where the open and closed arms join, has been difficult, particularly in mice, who can spend at least 20–30% of the test session in this region (Lee and Rodgers, 1990; Rodgers et al., 1992). Also, there is more continuous exploration of the EZM, as it eliminates the “boxed ends” inherent to the closed arms of the EPM. A test session of the EZM is 5 min in length; animals are placed at a randomly chosen boundary between an open and closed quadrant, facing the inside of the closed zone. Measures of interest include the time spent in the open quadrants, latency to enter an open quadrant, and the number of entries to open quadrants; these variables are sensitive to the effects of standard anxiolytic agents including benzodiazepines, zolpidem and phenobarbitone (Kulkarni et al., 2007).

In addition to the measurement of approach/avoidance behavior by assessment of activity in open and closed zones, both the EPM and EZM are amenable to the study of ethological behaviors such as head dips (downward movements of the head toward the floor) and stretch attenuated postures (elongation of the body with the feet remaining in place) (Shepherd et al., 1994; Rodgers et al., 1997). These directed exploration and risk-assessment behaviors are suggested to be less sensitive to potential locomotor confounds [but see (Weiss et al., 1998)] and may be related to the “apprehension and excessive vigilance” observed in clinical anxiety (Cryan and Holmes, 2005). Head dipping may be considered directed exploration (Weiss et al., 1998), and an increase in this behavior reflects decreased levels of anxiety. Stretch attenuated postures reflect risk-assessment, and increases in this behavior suggest heightened levels of anxiety. For example, the anxiolytic agent chlordiazepoxide was shown to increase the amount of time rats spent in the open quadrants of the EZM and frequency of head dipping, and decrease the number of stretch-attenuated postures (Weiss et al., 1998).

## Anxiety-Like Behaviors Following Experimental TBI

### Animal Models of Experimental TBI

The literature on rodent models of TBI becomes more voluminous by the year, with numerous experimental models of TBI available to investigators. A full description of these models goes beyond the scope of this paper, but the reader is directed to several reviews (Xiong et al., 2013; Johnson et al., 2015; Marklund, 2016; Povlishock, 2016; Bodnar et al., 2019; Ma et al., 2019). In brief, pre-clinical TBI models include considerably invasive approaches that require a craniectomy, such as controlled cortical impact (CCI) (Osier and Dixon, 2016) and fluid percussion injury (FPI) (Lyeth, 2016), which result in focal and combined focal/diffuse injuries, respectively. These models have been established for decades and have produced a wide body of literature. Although CCI and FPI have remained popular, in recent years attention has turned to less invasive and more clinically relevant “closed-head” models that produce a concussive, more diffuse injury, with or without acceleration/deceleration and rotational components (Bodnar et al., 2019; McNamara et al., 2020). Also of growing interest are animal models of blast overpressure (Skotak et al., 2019; McCabe and Tucker, 2020), as TBI from blast has been the signature wound from military conflicts for the past 20 years.

A significant and important point of discussion has been the distinction between mild, moderate and severe experimental TBI, both within and between different models. Within a given translational study, the severity of the injury as defined by injury device parameters can have effects of both behavioral and pathological outcomes (Saatman et al., 2006; Washington et al., 2012; Namjoshi et al., 2017; Tucker et al., 2017). Clinically, there have been clearly defined criteria for classifying TBI as mild, moderate or severe, employing measures including duration of loss of consciousness and post-injury amnesia, structural imaging, and the Glasgow Coma Scale (Cassidy et al., 2004; Management of Concussion/mTBI Working Group, 2009; Centers for Disease Control and Prevention, 2015). Although guiding principles for classification of TBI severity within the CCI model have been proposed using injury parameters (e.g., depth and velocity of impact) and outcomes such as tissue loss, neurological severity score, and cognitive deficits (Siebold et al., 2018), other variables such as injury location and whether the skull cap was replaced can contribute to injury severity. Furthermore, high-speed imaging of CCI devices has shown that there is variability in the operation between different devices, albeit modest (Kim et al., 2018). Similar issues exist for FPI and closed-head models; thus, it remains difficult to label and compare injuries as mild, moderate or severe.

Another important but difficult distinction is between acute and chronic effects of TBI in animal models. Clinically, the effects of mild TBI or concussion typically resolve within 7–10 days, with only a percentage of patients (10–25%) having symptoms persisting beyond 3 months; symptoms that can include neuropsychiatric complaints like anxiety and depression (Dwyer and Katz, 2018; Polinder et al., 2018). There is no agreement regarding the definitions of “acute” and “chronic” with respect to functional deficits in rodents following TBI (Osier et al., 2015). In a review of long-term deficits following TBI, Gold and colleagues defined “long-term” as 1 month or later, as they were interested in an optimal time point that would enable the determination of safety and efficacy of stem cell therapies (Gold et al., 2013). In a later review on chronic effects of TBI in rodents, Osier and colleagues acknowledged the 1-month time point of the earlier review, but reduced the time point to 2 weeks to be more inclusive (Osier et al., 2015). In a recent paper employing multiple measures of function and neuroimaging following concussion with acceleration/deceleration and rotational components, the authors concluded that a post-injury time point of 7–14 days, when the injury was inflicted approximately at 13 weeks of age in mice, was approximately equivalent to 24 months of recovery in a young adult post-concussion (Barretto et al., 2021). Thus, a time point in the range of 2 weeks to 1 month post-injury in rodents may be appropriate for modeling chronic symptoms observed clinically.

### Anxiety-Like Symptoms Post-TBI in Rodents

Despite the importance of testing for symptoms of anxiety in animal models of TBI, results have been woefully irreconcilable (Malkesman et al., 2013; Semple et al., 2019). Most often, to assay anxiety-related symptoms, a single behavioral test is performed following experimental TBI. For example, in the well-established and popular CCI model inflicted over parietal cortex, and using the well-validated EPM or EZM, conclusions have been inconsistent: many investigators have reported an increase in anxiety-like behaviors in rats and mice during these tests following CCI (e.g., Chauhan et al., 2010; Almeida-Suhett et al., 2014; Tchantchou et al., 2014), others have found a decrease in anxiety (often called increased risk-taking, impulsivity, or “behavioral disinhibition”) (e.g., Washington et al., 2012; Tucker et al., 2017), whereas sometimes no differences between injured and control groups are found at all (e.g., Watanabe et al., 2013; Sierra-Mercado et al., 2015). Reviewing the literature does not suggest that injury severity, when it can be compared, is a factor (Popovitz et al., 2019). The literature is voluminous and broad, even within a single injury model such as CCI, and there is a lack of standardization of injury methods as well as in behavioral testing paradigms.

Table 1 summarizes many recent studies employing unconditioned tests of anxiety in translational TBI studies. It is not meant to be all-encompassing, but to summarize studies cited in this paper and provide an update to previous reviews (Malkesman et al., 2013; Semple et al., 2019). A full systematic review has not been performed on pre-clinical studies of anxiety-related behaviors following TBI [but see (Semple et al., 2019) for an excellent summary]. The most consistency is with the FPI model, where increased anxiety-like behaviors are observed in most studies in both rats (Das et al., 2019; Dobrachinski et al., 2019; Fucich et al., 2019; Beitchman et al., 2020; Lapinlampi et al., 2020; Barretto et al., 2021) and mice (Tan et al., 2020; Tapp et al., 2020; Bhowmick et al., 2021), although in some studies this result is dependent on the time testing took place after injury. There is limited evidence from some CCI studies that results and conclusions from anxiety testing may depend on the time after injury at which behavioral testing takes place, or on the specific behavioral test employed (Tucker et al., 2017; Popovitz et al., 2019). Tucker and colleagues, for example, reported that following a severe CCI (but not mild CCI), mice showed increased thigmotaxis (indicative of greater anxiety) in the OFT for up to 3 weeks following injury; however, these same mice spent greater times in exposed, more brightly lit areas of the LDB and EZM at the same time points, suggesting decreased anxiety-like states (Tucker et al., 2017). In a similar repeated measures design study, Popovitz and colleagues demonstrated more time-dependent, rather than test-dependent results (Popovitz et al., 2019). Prior to 1 month following injury, mice with a moderate to severe CCI showed increased anxiety-like behaviors in the EZM and EPM, whereas after 5 weeks, decreased anxiety-like behaviors were measured in the EZM and OFT (Popovitz et al., 2019). Both of these studies illustrate the complexity of the changes in behavior and evolution over time in functional changes after TBI, and suggest that a comprehensive approach is necessary for drawing firmer conclusions regarding anxiety symptoms.

**TABLE 1:** Summary of tests for anxiety-like behavior and results from translational TBI studies.

**Behavior test**	**Injury model**	**Species and strain**	**Injury details**	**Testing details**	**Effect of TBI**	**References**
Open field	Controlled cortical impact	Sprague-Dawley male rats, 90 days old, 300–325 g	Parietal cortex, 6 mm diameter tip, 2.8 mm depth, 4 m/s velocity	Day 8 post-injury	No effect	de la Tremblaye et al., 2021
		Sprague-Dawley male rats, 5–6 weeks old	Parietal cortex, 3 mm diameter tip, 2.0 mm depth, 3.5 m/s velocity	Days 1, 7, and 30 post-injury	Reduced time spent in the center on days 7 and 30 (increased anxiety)	Almeida-Suhett et al., 2014
		C57 mice, male, 14 weeks old	Parietal cortex, 3 mm diameter tip, 2.0 mm depth, 2.5 m/s velocity	Day 67 post-injury	No effect	Islam et al., 2021
		C57 mice, female, 8–9 weeks old	Parietal cortex, 3 mm diameter tip, 1.5 mm depth, 6 m/s velocity	Days 1, 3, and 5 post-injury	No effect	Ritter et al., 2021
		C57 mice, 3 months old	Parietal cortex, 3.5 mm diameter tip, 1.5 mm (mild), 2.0 mm (moderate), or 2.5 mm (severe) depth, 5.25 m/s velocity	Day 21 post-injury	No effect	Washington et al., 2012
		C57 mice, male and female, 9–10 weeks old	Parietal cortex, 3 mm diameter tip, 1.5 mm depth, 4.5 m/s velocity	Days 1, 10, 20	Reduced distance traveled in the center for mice in the severe group (increased anxiety)	Tucker et al., 2017
		C57 mice, male, 8–12 weeks old	Parietal cortex, 3 mm diameter tip, 1.0 (mild) or 2.0 (severe) mm depth, 5.0 m/s velocity	Day 7	No effect	Lee et al., 2019
		C57 male mice, 6–8 weeks old	Parietal cortex, 3 mm diameter tip, 1.5 mm depth, 4.5 m/s velocity	Weeks 1, 3, 5 and 7 post-injury	Increased time spent in center zone at week 7 (decreased anxiety)	Popovitz et al., 2019
	Fluid percussion injury	Sprague-Dawley male rats, ∼350 g	3.25 ATM pressure	Days 29 and 127 post-injury	Reduced time spent in the center (increased anxiety) on Day 29	Lapinlampi et al., 2020
		Sprague-Dawley male rats, 250–300 g	2.5–3.0 ATM pressure	Day 35 post-injury	Reduced time spent in the center (increased anxiety)	Das et al., 2019
		Sprague-Dawley male rats, 279–420 g	2.19 ATM pressure	Days 7 and 28 post-injury	No effect of injury at Day 7; on Day 28 injured rats spent less time and made fewer entries into the center of the arena (increased anxiety)	Beitchman et al., 2020
		Wistar rats, male, 175–200 g	>2 ATM pressure	Day 7 post-injury	Reduced time spent in the center (increased anxiety)	Fucich et al., 2019
Light-dark box	Controlled cortical impact	C57 mice, male and female, 9–10 weeks old	Parietal cortex, 3 mm diameter tip, 1.5 mm depth, 4.5 m/s velocity	Days 2 and 21 post-injury	Reduced time spent in the dark chamber in “severe” TBI mice (decreased anxiety)	Tucker et al., 2017
		C57 mice, male, 8–12 weeks old	Parietal cortex, 3 mm diameter tip, 2.0 mm depth, 3.0 m/s velocity	Day 7 post-injury	No effect	Lee et al., 2019
	Fluid percussion injury	C57 mice, male, 8–10 weeks old	1.94 ATM pressure	Day 32 post-injury	No effect	O’Brien et al., 2021
		C57 mice, male, 9 weeks old	0.68 and 1.36 ATM pressure	48 h and 14 days post-injury	Decreased time spent in the light chamber (increased anxiety) and reduced number of transitions	Bhowmick et al., 2021
		Sprague-Dawley rats, male, 200–300 g	2.0 ATM pressure	1, 2, 3 and 4 weeks post-injury	Decreased time spent in the light chamber and fewer entries into light conditions (increased anxiety)	Barretto et al., 2021
Elevated plus maze	Controlled cortical impact	Sprague-Dawley rats, male, 300–350 g	Parietal cortex, 4 mm diameter tip, 1.5 mm depth, 5 m/s velocity	Days 2, 8, 15, and 29 post-injury	No effect	Tchantchou et al., 2021
		Swiss Webster mice, male, 6–8 weeks old	Parietal cortex, 3 mm diameter tip, 1.2 mm depth, 5 m/s velocity	Day 17 post-injury	Decreased entries to open arms (increased anxiety)	Karelina et al., 2021
		C57 male mice, 6–8 weeks old	Parietal cortex, 3 mm diameter tip, 1.5 mm depth, 4.5 m/s velocity	Weeks 1, 3, 5 and 7 post-injury	Decreased time in the open arms at Week 3 time point (increased anxiety)	Popovitz et al., 2019
		C57 mice, 3 months old	Parietal cortex, 3.5 mm diameter tip, 1.5 mm (mild), 2.0 mm (moderate), or 2.5 mm (severe) depth, 5.25 m/s velocity	Day 21 post-injury	All injured mice spent more time in the open arms (decreased anxiety)	Washington et al., 2012
		C57 mice, male, 2–4 months old	Parietal cortex, 3.0 mm diameter tip, 0.6 mm depth, 6 m/s velocity	Day 7 post-injury	No effect	Sierra-Mercado et al., 2015
		C57 mice, male, 2–3 months old	Parietal cortex, 3.0 mm diameter tip, 0.8 mm depth, 4.5 m/s velocity	Day 28 post-injury	No effect	Watanabe et al., 2013
		C57 mice, male, 3 months old	Parietal cortex, 3 mm diameter tip, 1.0 mm depth, 3.0 m/s velocity	Days 1 and 2 post-injury	Decreased number of open arm entries and open arm time on both Days 1 and 2 (increased anxiety)	Chauhan et al., 2010
	Fluid percussion injury	Rats, female, 4 months old	2.0 ATM pressure	Day 9 post-injury	No effect	Stielper et al., 2021
		Sprague-Dawley male rats, adult	3.25 ATM pressure	Days 28 and 126 post-injury	On Day 28, injured rats had longer latency to first entry to closed arm (decreased anxiety). On Day 126, injured rats made fewer entries and spent less time in open arms (increased anxiety).	Lapinlampi et al., 2020
		Wistar male rats, 120 days old, 280–320 g	1.55 ATM pressure	Day 14 post-injury	Injured rats spent less time in the open arms (increased anxiety)	Dobrachinski et al., 2019
		C57 mice, male, ∼10 weeks old	1.0–1.5 ATM pressure	Separate groups of animals tested 1 week or 12 weeks post-injury	Injured mice spent greater time in the open arms 1 week after injury (decreased anxiety). At 12 weeks, injured mice spent less time in the open arms (increased anxiety).	Tan et al., 2020
		C57 mice, male, 8–10 weeks old	1.94 ATM pressure	Day 33 post-injury	No effect	O’Brien et al., 2021
	CHIMERA	C57 mice, male, 8 weeks old	Repeated injury, once a day for 5 days, 0.5 J impact energy	Day 26 post-injury	Increased time spent in open arms (decreased anxiety)	Nolan et al., 2018
Elevated zero maze	Controlled cortical impact	C57 male mice, 6–8 weeks old	Parietal cortex, 3 mm diameter tip, 1.5 mm depth, 4.5 m/s velocity	Weeks 1, 3, 5, and 7 post-injury	Decreased time in open quadrants at week 1 (increased anxiety), increased time in open quadrants at week 5 (decreased anxiety)	Popovitz et al., 2019
		C57 mice, male, 14 weeks old	Parietal cortex, 3 mm diameter tip, 2.0 mm depth, 2.5 m/s velocity	Day 31 post-injury	Injured mice spent more time in the open quadrants (decreased anxiety)	Islam et al., 2021
		C57 mice, male and female, 9–10 weeks old	Parietal cortex, 3 mm diameter tip, 1.5 mm depth, 4.5 m/s velocity	Days 2 and 21 post-injury	Mice with severe injury spent less time in the darker quadrants (decreased anxiety)	Tucker et al., 2017
		C57 mice, male, 8 weeks old	Parietal cortex, 3 mm diameter tip, 1.5 mm depth, 5.0 m/s velocity	Days 6 and 14 post-injury	Time spent in the open quadrant per visit was decreased on day 14 (increased anxiety)	Tchantchou et al., 2014
	Fluid percussion injury	C57 mice, male and female, 8–10 weeks old	Pressure not given	Day 3 post-injury	Injured mice show preference for closed quadrants (increased anxiety)	Tapp et al., 2020

As stated prior, the CCI literature is broad. CCI is considered a focal injury, and unfortunately these inconsistencies in results extend to more diffuse and “closed-head” injury models (weight-drop, blast overpressure, and single or repeated concussive brain injury); further indicating that better standardization of TBI models and behavioral testing is needed. A newer TBI model, the Closed-Head Model of Engineered Rotational Acceleration (CHIMERA), a commercially available device that produces a contact force resulting in a rotational injury with acceleration/deceleration components, provides a more limited set of data on anxiety-like behaviors following experimental TBI (McNamara et al., 2020). To date, although more data are needed, results with the CHIMERA model have shown relative consistency: increased anxiety is measured in the OFT test up to a couple of weeks following injury, whereas decreased anxiety-like behaviors are found with the EZM or EPM at more chronic time points (e.g., Namjoshi et al., 2014; Nolan et al., 2018; McNamara et al., 2020). Future work with the clinically relevant CHIMERA model, employing multiple behavioral paradigms for anxiety and sophisticated analysis techniques will aid in the understanding of the development of symptoms of anxiety post-TBI, their biological underpinnings, and potential therapeutic targets.

### Consideration of Potential Testing Confounds

For any behavioral test following an experimental manipulation, it is critical to consider the animals’ ability to perform the behavioral task; sensorimotor function is especially important during performance in unconditioned tests of anxiety (Cryan and Holmes, 2005). Differences between groups in general arousal or activity may lead to perceived differences in levels of anxiety. For example, Algamal and colleagues found that mice subjected to two sessions of 21 days of repeated uncontrolled stress showed anxiety-like behavior in the EPM, which was ameliorated when the animals were also subjected to repeated mild TBI (Algamal et al., 2019). The authors suggested that this finding, as well as others in their study, should be interpreted carefully as increased locomotion, or “behavioral disinhibition,” from the mild TBI procedure (Algamal et al., 2019). This “impulsive” behavior has been described as increased time and entries to the open arms or quadrants of the EPM or EZM in injured rodents, and this could be related to changes in overall arousal due to TBI (Mannix et al., 2014; Mouzon et al., 2014; Gold et al., 2018; Tucker et al., 2019). Hyperactivity has been reported in multiple experimental models of TBI, including CCI (Kimbler et al., 2012; Budinich et al., 2013; Hsieh et al., 2014; Bajwa et al., 2016; Tucker et al., 2017) and repetitive concussive brain injury (Kane et al., 2012; Mannix et al., 2014; Tucker et al., 2019; Vu et al., 2020). Dissociating anxiety from activity and impulsivity has long been a point of discussion in translational anxiety research (Dawson and Tricklebank, 1995; Weiss et al., 1998; Cryan and Holmes, 2005). Suggestions for overcoming this confound include the measurement of ethological “risk assessment” behaviors including head dips and stretch attenuated postures (in the EPM and EZM) which may be less affected by overall arousal (Weiss et al., 1998; Cryan and Holmes, 2005), or the use of locomotor activity as a covariate in statistical analysis (Braun et al., 2011).

As unconditioned tests of anxiety partly rely on discrimination between light and darkness, the visual system plays a role in task performance. Deficits in visual acuity have been demonstrated following concussive, weight-drop, CHIMERA- and blast-induced TBI in rodents (Xu et al., 2016; Desai et al., 2020; Evans et al., 2021; Morriss et al., 2021; Tucker et al., 2021), but a basic light/dark discrimination ability may be intact and adequate for performance. Filgueiras and colleagues demonstrated that rats no longer avoid open arms of the EPM if they are enclosed with clear walls, suggesting the absence of physical walls is the anxiogenic factor (Filgueiras et al., 2014). Thus, perhaps of more importance than the visual system are vibrissae and somatosensory cortex; as an animal assesses the environment it prefers to keep its vibrissae in contact with walls (Prut and Belzung, 2003; Crawley, 2007). In an OF environment, mice without vibrissae will no longer show thigmotaxis (Prut and Belzung, 2003), although a lack of vibrissae alters primary EPM measures little in rats, suggesting compensatory mechanisms are in place (Filgueiras et al., 2014) [but see (Belzung, 1999) for observations in mice].

### Repeated Testing in Unconditioned Tests of Anxiety

Baseline measures prior to experimental manipulation, and/or testing at multiple time points during the experiment, may be desirable or necessary. However, performance during subsequent exposures to a behavioral test is affected by prior experience with the apparatus and environment, and the change may be treatment-dependent. Activity in the center has been shown to be relatively consistent across multiple trials (Bouwknecht et al., 2004), but the majority of behavioral data regarding OFT habituation relates to overall activity or locomotor activity in the apparatus. Hall initially described a decrease in the amount of locomotor activity in the OFT in rats with each subsequent exposure, reflective of a habituation process (Hall, 1934). Since that time, between-session habituation with repeated OFT testing has been confirmed by many in rats (Cerbone and Sadile, 1994; Dubovicky and Jezova, 2004; Haleem et al., 2015; Poveda et al., 2020) and mice (Crusio and Schwegler, 1987; Bolivar et al., 2000; Sturman et al., 2018; Rudeck et al., 2020), and is considered to be one of the simplest forms of hippocampal-dependent non-associative learning (Cerbone and Sadile, 1994; Leussis and Bolivar, 2006). To avoid this potential confound, some investigators choose to test in the OFT only once, comparing experimental groups in their response to a novel environment (Washington et al., 2012; Breu et al., 2016; Islam et al., 2021). Rudeck and colleagues recently applied repeatability analysis to data acquired for 5-min sessions in the OFT for seven consecutive days, in three strains of mice (C57BL/6J, BALB/cJ, and 129S1/SvlmJ) (Rudeck et al., 2020). The authors concluded that a 3 day habituation period is sufficient to establish a stable pattern of distance traveled, although activity in the center of the apparatus was not measured. Furthermore, the variance in the data could not be explained by mouse strain or individual animal in the first 3 days of testing, and was attributed by the authors to unknown factors such as stress or anxiety (Rudeck et al., 2020). In the following days, individual differences between mice, or “personality,” was found to be the primary contributor to the variance in exploratory behavior (Rudeck et al., 2020). Although a 3-day habituation with 5-min sessions was suggested, it is unclear if a single 15-min session would have the same effect.

With repeated testing, mice may also habituate to the LDB apparatus. They learn to locate the position of the opening between the light and dark chambers and the latency to transition from the light to the dark decreases with repeated exposures (Barnes et al., 1990). However, this requires multiple trials. Holmes and colleagues showed stable baseline behavior with two exposures to the LDB (Holmes et al., 2001); Blumstein and Crawley exposed mice to six trials over a 2 week period, and found that the number of transitions between the light and dark compartments remained stable for the first three trials, and this result was independent of inter-trial interval (ranging from 1 to 7 days) (Blumstein and Crawley, 1983). A similar study, measuring exploratory activity, found that repeated daily testing was possible for 4 days (Onaivi and Martin, 1989). Accordingly, Bouwknecht and colleagues performed four LDB trials at 1-week intervals in mice, and reported little change over those four trials in the number of transitions between the light and dark chambers, the time spent in the dark chamber, and the latency to enter the dark compartment (Bouwknecht et al., 2004). Thus, it appears that stable results may be obtained for up to four trials in the LDB.

There is a wide body of literature on repeated testing in the EPM. Briefly, initial EPM experiments suggested that behavior changed little with repeated exposure to the maze (Pellow et al., 1985; Lister, 1987). Since that time, however, the “one-trial tolerance” phenomenon has been described in which after a first exposure to the EPM, open arm exploration is significantly decreased in rats and mice, and anxiolytic agents are no longer effective at increasing the amount of time in the open arms (File et al., 1990; Rodgers et al., 1992; Rodgers and Shepherd, 1993; Treit et al., 1993; Holmes and Rodgers, 1998, 1999; Zhou et al., 2015; Bourin, 2019) [but see (Schrader et al., 2018)]. There are multiple proposed explanations for the one-trial tolerance phenomenon, discussion of which goes beyond the scope of this paper [but see (Bourin, 2019) for review]. However, it is widely agreed that the EPM is typically an unsuitable assay for longitudinal studies that require multiple trials.

The EZM may be a better choice for experimental designs that require testing at multiple time points. In a direct comparison of the EPM and EZM under identical laboratory conditions, Tucker and McCabe demonstrated, in mice, that while overall activity (distance traveled) and time spent in the open regions of the apparatus decreased significantly after one trial in the EPM, these measures remained stable for at least three trials in the EZM, regardless of inter-trial interval (weekly or daily) (Tucker and McCabe, 2017). Stability of EZM behavior has also been demonstrated in rats with a testing interval of 1–2 months (Ajao et al., 2012; Kamper et al., 2013), or with a daily interval for four trials (Blokland et al., 2012). Cook and colleagues, however, found increased anxiety-like behaviors in mice following a single exposure to the EZM, though their testing conditions varied from other studies (Cook et al., 2002).

## Future Directions of Behavioral Testing in TBI Research

### Use of Multiple Tests and Alternative Data Analysis Methods

In addition to better reporting of injury and testing parameters, broader testing and alternative data analysis methods may aid in the assessment of anxiety. As mentioned prior, the majority of translational TBI studies employ only one unconditioned test of anxiety at a single time point to assay anxiety-like behavior. Many experts in the field of rodent behavioral testing suggest a test battery (Cryan and Holmes, 2005; Ramos, 2008). However, a test battery presents with its own set of interpretation difficulties. As described by Ramos, “we need to test it in different (behavioral) models, but by doing so at different times, we would never know, for example, whether an animal seemed fearful in the EPM and brave in the OF because of its fluctuating mood or because of construct differences between tests” (Ramos, 2008). Testing order and interval between tests are critical decisions when employing multiple tests; testing order is recommended to proceed from least stressful to most stressful. The OFT has been shown to be a relatively non-stressful test (Bodden et al., 2018), and a proper testing order for three unconditioned anxiety tests has been suggested as OFT, LDB, and last, EZM or EPM, with at least a 48-h interval between tests (Tsuda et al., 2020). Employing a combination of conditioned and unconditioned tests has also been suggested (Pentkowski et al., 2021).

Recognizing that only a subgroup of TBI patients are affected by anxiety, recent studies in the TBI literature have highlighted the usefulness of expanding data analysis methods beyond group mean comparisons (Scholten et al., 2016). Whereas clinical studies have rigorous inclusion criteria, most pre-clinical studies assessing anxiety after TBI consider the injured group as a homogeneous distribution, rather than considering variability among individuals and using that individual variability following TBI to learn about underlying pathology. Popovitz et al. tested male mice on a battery of anxiety tests (EZM, OFT, EPM) at multiple time points following CCI (weeks 1, 3, 5 and 7) and developed a multi-dimensional behavioral profiling technique to identify “resilient” and “vulnerable” subgroups of mice (Popovitz et al., 2021). The authors reported that only about 13% of the injured animals were found to be “vulnerable,” showing increased exploration of anxiogenic regions during testing compared to baseline behavior and to sham-treated animals (Popovitz et al., 2021). The behavior of the vulnerable mice had neurobiological correlates in the medial prefrontal cortex, basolateral amygdala, and ventral hippocampus; all areas that are associated with stress and anxiety (Almeida-Suhett et al., 2014; Bryant and Barker, 2020; Kenwood et al., 2021; Liu et al., 2021; McCorkle et al., 2021; Pentkowski et al., 2021).

Statz et al. employed a different “affective profiling” technique to identify “affected” and “unaffected” rats 3 weeks or 6 months following exposure to repeated blast overpressure (Statz et al., 2019). Functional outcomes were assessed in the LDB, EZM, response to fear conditioning, and molecular markers included plasma corticosterone (CORT) and stathmin-1 (a protein associated with microtubule assembly that has been shown to be elevated in the amygdala following TBI, and is elevated when there are increased levels of fear in rodents). Approximately 30–40% of the injured rats were identified as “affected,” with increased anxiety-like behaviors and/or protein levels different from control animals. As evidenced by elevated plasma CORT levels in affected animals at that time point, the stress response was associated with anxiety-related behaviors at 3 weeks following injuries, but not at 6 months. Amygdalar stathmin-1 levels were elevated at 3 weeks, whereas prefrontal cortex stathmin-1 levels were decreased at 6 weeks post-injury in affected rats (Statz et al., 2019). This study provides further evidence that behavioral measures in “affected” or vulnerable animals can correlate with biological measures in the amygdala and/or measures that suggest heightened levels of stress.

### Employment of Common Data Elements for Preclinical Anxiety Studies

Preclinical assessment of anxiety symptoms in the laboratory setting has considerable challenges. As previously noted, the literature on a particular behavioral test suggests inconsistent findings between laboratories are inexplicable; questioning the utility of a particular test and its validity for the study of anxiety in preclinical models of TBI. In part this derives, however, from the many factors that affect behavioral performance, including the animal’s phenotype, species, strain, sex, age, and housing conditions, the nature of the TBI model (level of severity, consequent neuropathology, additional factors such as peripheral sites of trauma), and testing conditions [time of day, room settings, dimensions of test apparatus, handling, test sequences, and the particular chosen behavioral measure(s)]. Certain variables are impossible to control due to inherent properties of the test system’s (i.e., animal’s) biology and interaction of a particular species with the physical properties of the test system. But many of the variables may be controllable—permitting improved cross-study comparability—by standardization of procedures across laboratories; much as in clinical studies where there are great efforts expended to employ shared procedures at member clinical test sites. Yet, it has been a long-time discussion regarding the feasibility, and perhaps futility, of promulgating and “enforcing” standards for testing apparatuses, test room conditions, and the precise behavioral measures (Wahlsten, 2011).

In addition to early discussions regarding preclinical reproducibility (Landis et al., 2012; Collins and Tabak, 2014) and inclusion of both sexes (Clayton and Collins, 2014), the development of computer technology, informatics, and machine learning has been an impetus that led researchers to an alternative view. Within reasonable parameters, individual investigators can utilize conditions for which they have established standard procedures locally. The key for progress is seen with *reportage* at a level that allows studies to have greater comparability, permitting higher analyses to take place by the accumulation of data, by comparable links, across individual studies. For evaluating the effects of TBI and treatment for anxiety, there are key elements that can be carefully documented for the animal phenotype, the TBI model, and the behavioral test(s) employed for measuring anxiety. An initiative spearheaded by the National Institute for Neurological Disorders and Stroke began over a decade ago to develop *common data elements* (CDEs) that could be ascribed as essential reportable information for clinical research that would permit data sharing, collaboration, and eventual and more efficacious comparisons across studies (see Thurmond et al., 2010; Hicks et al., 2013 for descriptions of the earliest developments), with general descriptions of variables that described research participant assessment outcomes across a number of functional domains (Wilde et al., 2010). This led to efforts to expand the practice to preclinical TBI research, where the development of a “common language” could facilitate combining data sets based upon improved and more detailed description of the TBI preclinical model used for the data collection by individual laboratories (Smith et al., 2015). The goal was to define and standardize, not how individual studies were executed, but to have a system that permitted comparability across studies by application of accepted data elements. To initiate the effort, the Federal Interagency Traumatic Brain Injury Research (FITBIR) Informatics System was created as an informatics system and data repository for Preclinical Traumatic Brain^1^ Injury (TBI) research.

Employment of the platform has as its first step the application of definitions for each pertinent independent and dependent variable relevant to a range of variables or data about the nature of the study, the characteristics of the animals employed, the injury model, and individual measures related to outcome, such as behavioral tests. For preclinical studies this would involve description of the *core CDEs*, i.e., the characteristics of the animals, pertinent information related to application of the injury model, characteristics of the device employed, and assessments and outcomes (c.f., Smith et al., 2015). A major advancement in application was the subsequent review of the original effort and the development of 913 CDEs by a TBI Preclinical Working Group that has formulated critical data elements (LaPlaca et al., 2021). The Working Group then organized the CDEs into logical groups and 46 Form Structures, that broadly organized the CDEs into a Main Group, Animal and Study Data groups, Injury Models, and Assessments and Outcomes that includes 1664 CDEs (cf., Tables 1, 2 in LaPlaca et al., 2021). The FITBR site has website links to formally document animal phenotype, TBI model, as well as behavioral readouts.^2^ Pertinent to preclinical studies for anxiety assessment, there are specific links for the OFT, the EZM, and EPM (Table 2). There are presently no postings for the LDB; Table 3 provides an outline that parallels the Form Structures formulated by the Working Group (LaPlaca et al., 2021).

**TABLE 2:** Common data element Links at FITBR for Preclinical Study of TBI and Anxiety.

**CDE Group**	**No. Elements**	**Short Description**	**Key Reference/Example**	**Cdc.nlm.nih.gov.link**
Main Group*	88	General information about study, animal characteristics, injury*	#Table 1 (Smith et al., 2015), Table 1 (LaPlaca et al., 2021)	http://www.ncbi.nlm.nih.gov/pubmed/26058402 https://www.ncbi.nlm.nih.gov/pubmed/33297844
Animal Characteristics	83	Species, strain, age, genetics, sex, vendor	#Table 1 (Smith et al., 2015), Table 1 (LaPlaca et al., 2021)	http://www.ncbi.nlm.nih.gov/pubmed/26058402 https://www.ncbi.nlm.nih.gov/pubmed/33297844
**TBI Model**				
CCI	29	Surgery and device descriptors, impactor type and settings, craniectomy	#Table 2 (Smith et al., 2015)	https://cde.nlm.nih.gov/formView?tinyId=7yeWOg_x
FPI	32	Surgery and device descriptors, peak pressure and settings, craniectomy	#Table 2 (Smith et al., 2015)	https://cde.nlm.nih.gov/formView?tinyId=QsDZ8Ltd
Blast	66	Device descriptors, driver gas, pressure descriptors, body exposure and orientation	#Table 1 (Rodriguez et al., 2018), #Table 2 (McCabe and Tucker, 2020)	https://www.ncbi.nlm.nih.gov/pubmed/29160141 https://cde.nlm.nih.gov/formView?tinyId=wn_HMj65
CHIMERA	20	Device descriptors, impact variables; linear/angular velocity and acceleration (g), head displacement	#Table 7 (McNamara et al., 2020)	http://www.ncbi.nlm.nih.gov/pubmed/32692987
Weight Drop		Surgery and device descriptors, weight drop height, mass	#Table 2 (Smith et al., 2015)	http://www.ncbi.nlm.nih.gov/pubmed/26058402
Projectile Impact		Surgery and device descriptors, contact pressure	#Table 2 (Smith et al., 2015)	http://www.ncbi.nlm.nih.gov/pubmed/26058402
**Behavioral Readouts**				
Open Field Test	69	Behavioral measures, equipment descriptors, scoring/software, room environment, acclimation, injury elapsed time		https://cde.nlm.nih.gov/formView?tinyId=7JB1cozsN
EPM	35			https://cde.nlm.nih.gov/formView?tinyId=7JgyTujfoN
EZM	35			https://cde.nlm.nih.gov/formView?tinyId=Xyz1KoMj4
Light-Dark Box	10		See Table 3	

*Many of these features also appear in other Form Structures described below Main Group. #These publications are available as Open Access. https://cde.nlm.nih.gov/form/search?selectedOrg=NINDS&classification=Preclinical%20TBI.

**TABLE 3:** Common data elements for light-dark box (LDB) documentation.

**CDE Group**	**Short Description**
Main Group*	General information about study, animal characteristics, injury
Animal Characteristics*	Species, strain, age, genetics, sex, vendor
**Behavior Measure**	
Device Description	Device dimensions, vendor
Acclimation	Duration of acclimation to test room
Illumination	Illumination in light and dark chambers
Software	Software, vendor for behavior measures
Test Duration	Total time animals in light and dark chambers
Time in compartments	Total duration animal spends in light and dark chambers
Latency to Transition	Latency to make initial movement after placement in box
Transitions	Number times animal crosses between light and dark chambers
Head Pokes	Number of times animal pokes head into light chamber
Limb entries	Number of forelimb and hindlimb entries to light chamber

*See links provided in Table 2.

As noted, a prime goal for application of preclinical CDEs in TBI research would enhance rigor and reproducibility, as well as providing the details and procedures of each published research report, with an endpoint for better relating findings with clinical TBI phenotypes. While this may not resolve all incongruities in findings across laboratories, it is seen as a way forward for standardized and universal data collection for improvement of data quality and sharing.

## Summary and Conclusion

In summary, it is difficult to make broad conclusions regarding anxiety-like states in rodents following experimental TBI. The employment of unconditioned tests has led to a wide body of literature, yet disparate results for many rodent TBI models, across multiple tests. Unconditioned tests have been criticized for their inability to discriminate between locomotion, exploration and anxiety, yet their use will continue due to their ease of use and high-throughput.

Of course, the ultimate goal is to develop treatments for anxiety disorders post-TBI. Drug discovery for psychiatric disorders is complex; the use of single behavioral tests has been of limited use in developing approved treatments for anxiety and a more complete research program with multiple tests is more often required (Cryan and Sweeney, 2011). In this context, it should be noted that OFT results should be considered preliminary in the context of anxiety research (Crawley, 2007). It has been shown that some anxiolytic agents do not increase the time spent in the center of the OFT, demonstrating this measure to have low predictive validity (Heredia et al., 2014; Thompson et al., 2015), and it is suggested that OFT results should be followed up with anxiety-specific assays such as the LDB, EZM and/or EPM (Crawley, 2007). Additional tests that are less dependent on motor output, such as the Vogel conflict test (Millan and Brocco, 2003) and stress-induced hyperthermia (Olivier et al., 2003; Adriaan Bouwknecht et al., 2007), may also be useful additions to a battery of anxiety tests following experimental TBI. Standardization of injury and behavioral techniques, comprehensive testing, analyses that identify dysfunctional anxiety states in “affected” individual animals, and correlations between behavior and neurobiological markers can add to the value of rodent models of TBI.

## Author Contributions

LT conducted the literature review and writing. JM contributed to discussion and final editing. All authors contributed to the article and approved the submitted version.

## Author Disclaimer

The opinions, interpretations, conclusions and recommendations are those of the authors and are not necessarily endorsed by the U.S. Army, Department of Defense, the U.S. Government or the Uniformed Services University of the Health Sciences. The use of trade names does not constitute an official endorsement or approval of the use of reagents or commercial hardware or software. This document may not be cited for purposes of advertisement.

## Conflict of Interest

The authors declare that the research was conducted in the absence of any commercial or financial relationships that could be construed as a potential conflict of interest.

## Publisher’s Note

All claims expressed in this article are solely those of the authors and do not necessarily represent those of their affiliated organizations, or those of the publisher, the editors and the reviewers. Any product that may be evaluated in this article, or claim that may be made by its manufacturer, is not guaranteed or endorsed by the publisher.

## Footnotes

1
https://fitbir.nih.gov/content/preclinical-common-data-elements

2
https://cde.nlm.nih.gov/form/search?selectedOrg=NINDS&classification=Preclinical%20TBI
